# Identification of messenger RNA of fetoplacental source in maternal plasma of women with normal pregnancies and pregnancies with intrauterine growth restriction

**Published:** 2012-09-30

**Authors:** Paola Ayala Ramírez, Reggie García Robles, Juan Diego Rojas, Martha Bermúdez, Jaime Bernal

**Affiliations:** a Department of Obstetrics and Gynecology Pontificia Universidad Javeriana-Hospital Universitario San Ignacio, Bogotá, D.C., Colombia. And Gynecologist physician. Unit of Feto-maternal MedicineSS.E-mail: jdiegorojas@yahoo.com; b Instituto de Genética Humana, Pontificia Universidad Javeriana, Bogotá, D.C., Colombia. E-mail: rgarcia@javeriana.edu.co

**Keywords:** RCIU, Messenger RNA, plasma, pregnancy complications, human placental lactogen, Chain Reaction Reverse Transcriptase Polymerase

## Abstract

**Objective:**

to quantify placenta-specific RNA in plasma of women carrying foetuses with intrauterine growth restriction and pregnant women with normal pregnancies.

**Methods:**

8 pregnant women with foetuses with intrauterine growth restriction were studied as well as 18 women with uncomplicated pregnancies in the third pregnancy trimester. Total free RNA was quantified in maternal plasma by spectrophotometry and the gene expression of hPL (Human Placental Lactogen) at the messenger RNA level through technical Real Time-Chain Reaction Polymerase.

**Results:**

plasma RNA of fetoplacental origin was successfully detected in 100% of pregnant women. There were no statistically significant differences between the values of total RNA extracted from plasma (*p*= 0.5975) nor in the messenger RNA expression of hPL gene (*p*= 0.5785) between cases and controls.

**Conclusion:**

messenger RNA of fetoplacental origin can be detected in maternal plasma during pregnancy.

## Abstract (es)

**Objetivo:**

cuantificar RNA específico de placenta en el plasma de mujeres con embarazos con fetos con Restricción de Crecimiento Intrauterino y gestantes con embarazos normales.

**Métodos:**

se estudiaron 8 mujeres con embarazos con fetos con Restricción de Crecimiento Intrauterino y 18 mujeres con embarazos sin complicaciones, en el tercer trimestre de embarazo. Se cuantificó el RNA total libre en plasma materno por espectrofotometría y la expresión del gen hPL (Lactógeno Placentario Humano) a nivel de RNA mensajero por medio de la técnica Reacción en Cadena de la Polimerasa en Tiempo Real.

**Resultados:**

se logró detectar RNA en plasma de origen fetoplacentario en el 100% de las gestantes. No se encontraron diferencias estadísticamente significativas entre los valores de RNA total extraído de plasma (*p*= 0.5975) ni en la expresión del RNA mensajero del gen hPL (*p*= 0.5785) entre casos y controles.

**Conclusión:**

es posible detectar RNA mensajero de origen fetoplacentario en plasma materno durante el embarazo.

## Introduction

Intrauterine Growth Restriction (IUGR) is defined as the clinical diagnosis wherein the foetuses do not reach its full growth potential and the final outcome is a decrease in body weight, being below the 10th percentile for gestational age and sex according to growth charts^1^. The infant may have a small complexion or because of factors in the mother, in the placenta or foetuses ^2^. IUGR is a major burden on perinatal and neonatal morbidity and mortality^1^. In addition, children with IUGR subsequently may evolve with changes in growth and neurocognitive development^3^. The association of low birth weight (LBW) with subsequent development of adult diseases such as insulin resistance and cardiovascular disease has also been studied, this finding has been termed ''fetal programming''^4^. Biomarkers in maternal serum have been shown to have predictive power and sub-optimal sensitivity for fetal growth assessment^5^. Due to the limitations of current techniques, it is necessary to develop more accurate methods for monitoring fetal growth^6^. In 1997 Lo *et al*., managed to isolate free DNA (deoxyribonucleic acid) circulating in plasma of pregnant women^7^. These studies opened a new possibility of noninvasive prenatal diagnosis where neither the foetuses nor the mother are in any danger^8^. Because it is often not possible to determine the source of DNA found in plasma of pregnant women and can be confused with the maternal DNA, the question of whether RNA could be isolated from fetal-placental origin in maternal plasma and thus determine the expression of specific genes in the placenta was formulated. Poon *et al*., were the first to report the presence of fetal RNA in the plasma of pregnant woman^9^. Numerous studies have analyzed the gene expression at level of messenger RNA (mRNA) extracted from maternal plasma in fetal pathologies such as trisomy^10^ ^,^ ^11^, congenital cardiopathies^12^ and maternal complications such as: preeclampsia^13^ ^,^ ^14^, RCIU^6^ and previous percreta placenta^15^, stating that the use of genetic markers at the level of mRNA of fetal-placental origin circulating in maternal plasma opens up new possibilities for strategies in early detection of fetal pathological conditions, noninvasive prenatal diagnosis, determination of risk for developing complications of pregnancy and pathology severity. Human placental lactogen (hPL) is produced in the placenta and acts as an immunosuppressant inducing tolerance and gestational fetal growth factor^2^. Previous studies have linked low levels of mRNA in maternal plasma of hPL gene in pregnancies complicated by previous placenta^15^ and preeclampsia^14^ ^,^ ^16^. These studies conclude that real time-PCR is a sensitive method to monitor changes in mRNA levels resulting from apoptotic effects in the placenta and to evaluate the conditions of villous trophoblast invasion^15^. The aim of this study was to quantify total RNA by spectrophotometry and hPL gene expression at the level of free mRNA in maternal plasma by RT-qPCR technique in pregnancies that progressed with IUGR and pregnant women with uncomplicated pregnancies and normal foetuses.

## Materials and Methods

An analytical study was conducted in pregnant women in third trimester of pregnancy whose pregnancies progressed with IUGR and in pregnant women with uncomplicated pregnancy healthy foetuses, assisted by the Department of Gynecology and Obstetrics, of the Hospital Universitario San Ignacio in Bogotá between November 2009 and December 2010. Inclusion criteria were patients with diagnosis by conventional ultrasound or color Doppler ultrasound in fetal, placental and both uterine arteries of IUGR (weight less than 10 percentile for gestational age) and with no apparent etiology, we excluded patients with ultrasonographic diagnosis of IUGR and small newborn for gestational age with known cause, foetuses / newborn with major fetal malformations or diagnostic impression of genetic disease, maternal prothrombotic conditions and pregnant women who expressed their wish not to participate in the study. 

The study was approved by the Committee for Research and Bioethics of the Medical Faculty of Pontificia Javeriana University and fulfilled the guidelines established in the Helsinki declaration of 1975 as amended in 2004. All participants filled out a voluntary informed consent.

### Sample taking and processing:

Two samples of peripheral blood by venipuncture (5 cc) in tubes containing EDTA anticoagulant were taken. The samples were immediately centrifuged at 3,000 rpm for 15 minutes and the plasma (supernatant) was transferred to a 15 mL Falcon tube. 1.6 mL of plasma was mixed with 2 mL of Trizol LS (Invitrogen) and stored at -40 °C until processed. 

### RNA extraction:

extraction was performed in plasma using the protocol recommended by Ng *et al*.^17^ adding 5U of DNAsa (Epi-centre). Total RNA was stored at -40 °C. The total RNA concentration was determined at 260 nm using GeneQuant Pro (Amersham Bioscience).

Reverse transcription and PCR in Real time (qPCR-RT): reverse transcription of total RNA to cDNA was performed using the SuperScriptIII kit (Invitrogen, Carlsbad, CA, USA) in a reaction with 20 uL final volume containing 1X buffer, 0.5 mM dNTPs, 50 ng of random primer, 0005 M DTT, 40U of RNase OUT, 200U of SuperScript and 7 uL of RNA, with subsequent digestion with RNaseH 2U, following the manufacturer's recommendations. The cDNA was stored at -20°C until amplification. LigthCycler (Roche) thermal cycler was used using SYBR Green chemistry (LigthCycler FastStart DNA Master SYBR Green I kit, Roche) and the Lightcycler 4.1 software for absolute quantification (Roche Diagnostics). PCR was performed in a reaction volume of 20 uL with 7 uL of sample and 0.25 uM primer, following the manufacturer's recommendations. The program conditions of the PCR are shown in Table 1. The sequences of the primers were: 5´CATGACTCCCAGACCTCCTTC3' (sense) and 5'TGCGGAGCAGCTCTAGATTG3' (antisense); it confirmed that these primers do not amplify DNA with the Primer software^18^ and performing DNA amplification which did not show any fragment. The standard curve was performed as of the PCR product of purified hPL with the Wizard(r) SV Gel kit and PCR Clean-Up System (Promega), the number of copies was calculated according to Overbergh *et al*.^19^. Serial dilutions were performed with a range from 1X107 to 1X101 copies. Each sample was analyzed in duplicate and each analysis was mounted in a negative control in which cDNA instead of water was placed.

**Table 1:**
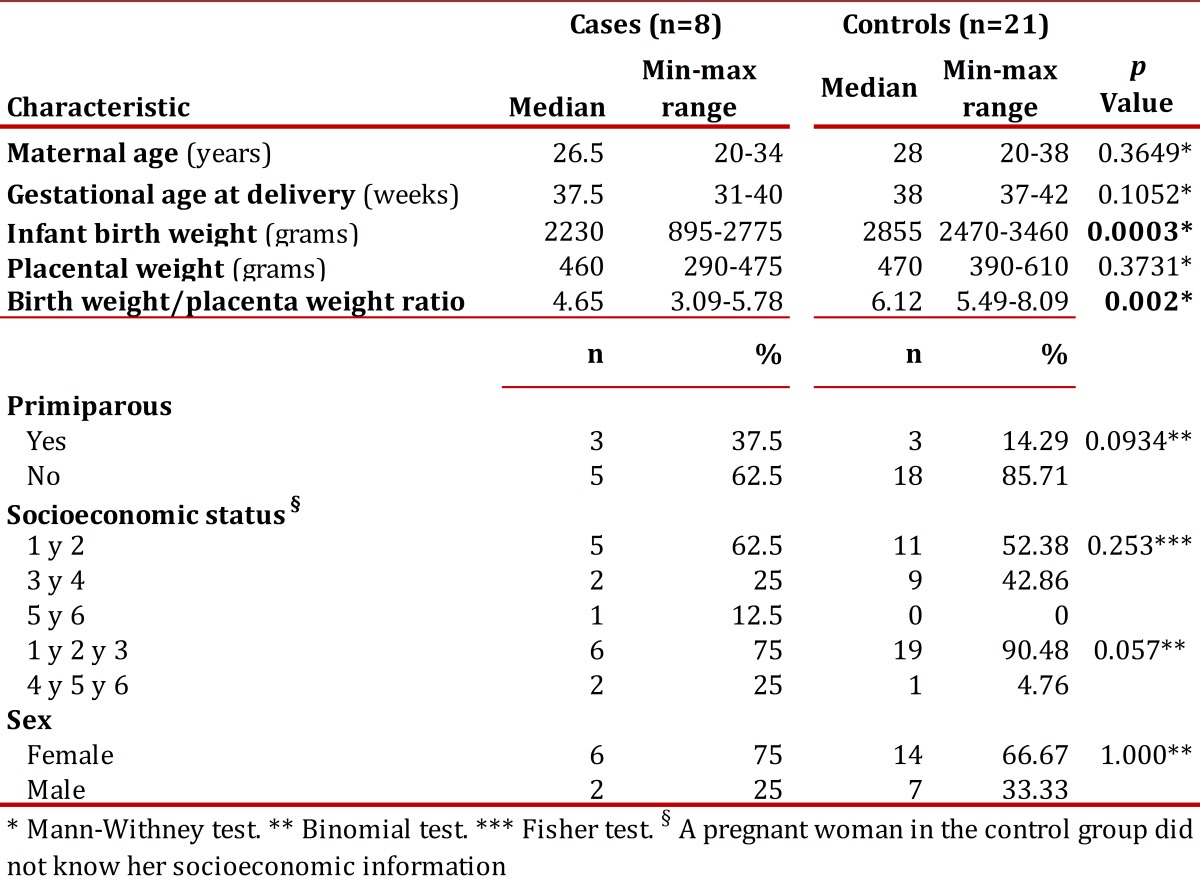
PCR amplification conditions of hPL gene

### Statistical Analysis:

analysis was performed by calculating median and ranges. We performed the hypothesis testing approach to determine whether there is association between the levels of total RNA and mRNA of hPL in plasma of normal controls and patients with IUGR using the Mann-Whitney test with Stata 9.1. 

## Results

In total, the studied collected data from 12 cases and 22 controls, but excluded 4 cases and 1 control because the newborn had some exclusion criteria. The case group was comprised of 8 women pregnant in the third trimester of pregnancy with a diagnosis of idiopathic IUGR. Table 2 shows the demographic characteristics of the population. The variables of birth weight and birth weight / placental weight index showed statistically significant differences (*p*= 0.0003 and *p*= 0.002, respectively). With respect to the classification of IUGR, 50% of cases had an asymmetric IUGR and 50% had symmetric IUGR. Additionally, 3 patients had mild IUGR, 5 moderate to severe and 2 patients had abnormal Doppler. 

**Table 2:**
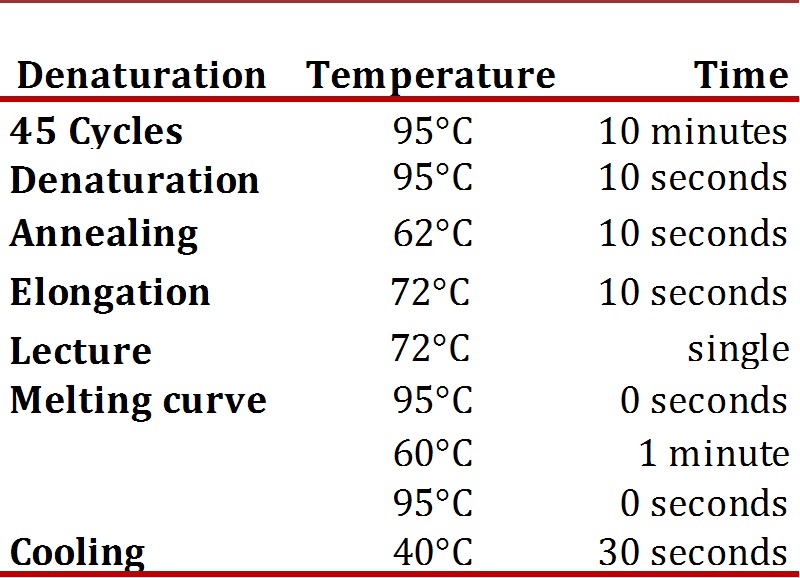
Demographic characteristics of the population

RNA extract was achieved from 100% of plasma samples. The results of the quantification of total RNA from plasma and hPL mRNA expression are described in Table 3. There were no statistically significant differences in the concentration of total RNA or mRNA expression of hPL (*p*= 0.5975 and 0.5785, respectively). 

**Table 3.**
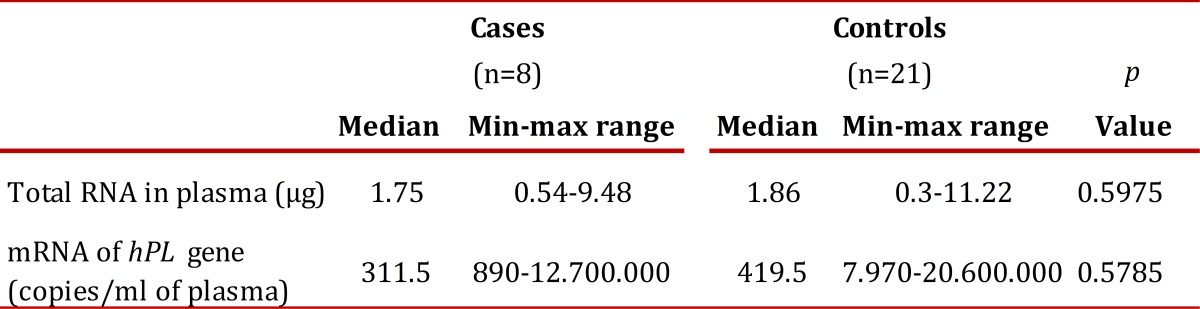
Quantification of total RNA and absolute quantification of hPL gene in pregnant women with foetuses with IUGR and normal pregnancy.

## Discussion

In this study, RNA was extracted from plasma of 100% of the samples and it was possible to quantify mRNA expression of the gene hPL by real time-PCR. These findings reproduce the results previously reported in the literature which shows that the placenta is an important organ that releases fetal RNA in maternal plasma^17^ ^,^ ^20^ ^,^ ^21^. The amount of total RNA obtained from plasma in controls is lower than that reported by Maron *et al*., who obtained plasma RNA levels between 3 and 780 mg of 3 individuals in the third quarter ^22^, however it should be noted that Maron et al study took 10 cc of whole blood and in this study we worked with 5 cc. The median levels of hPL gene mRNA in controls are higher than those reported by Tsui *et al*.^23^ (n= 5), Chiu *et al*.^20^ (n= 6), Okazaki *et al*.^21^ (n= 75) and Ng *et al*.^17^ (n=10), who reported levels in copies /ml of 14,707, 9,900, 3,698 and 122,225 (data inferred from the article) respectively and less than the data reported by Farina *et al*.^14^ (n= 30) who reported a median of 2,754,862. No statistically significant differences in levels of total RNA and mRNA hPL gene between cases and controls were found. 

This is the first study which evaluated the mRNA levels of hPL gene in patients with a diagnosis of idiopathic IUGR. In patients with preeclampsia have been found that mRNA levels of hPL gene are significantly lower in both pregnant in the first quarter^14^ as in the second and third trimesters^16^, these studies show that it is possible to monitor placental status in maternal plasma. The absence of statistically significant differences in our study could be due to sample size, so it is recommended expanding it. Another possibility could be that the absence of differences in placental weight between the groups and because hPL is a marker of placental weight, were not significant differences. The results of hPL protein expression in placentas with IUGR show no statistically significant differences with healthy placentas. Although it has been reported a decrease in protein levels of hPL in the plasma of women with significant IUGR differences^5^, we were unable to find these differences at the level of mRNA, which could be due to the slow release of mRNA compared to protein-fetal placental origin. Moreover, development of placental mRNA markers that can be detected in plasma represents a breakthrough in the use of molecules that could be used in all pregnant women regardless of fetal sex using DNA limiting factor. Furthermore Zong *et al*., hypothesizes that the quantitative assessment of fetal DNA in maternal plasma together with the measurement of mRNA from different transcripts could help improve identification of women at risk of developing preeclampsia^24^, which may also be proposed for IUGR.

## Conclusions

mRNA of fetoplacental origin was detected in plasma of colombian pregnant women in third trimester of pregnancy. The data presented demonstrate that it is possible to profile gene expression in the placenta by analyzing maternal plasma. The origin and release mechanisms of these molecules have not been completely elucidated and their understanding is likely to generate knowledge about the pathophysiology of diseases such as preeclampsia and IUGR. Additional studies are needed measuring mRNA levels in plasma of genes involved in the pathophysiology of the disease evaluated in normal and abnormal pregnancies to establish the feasibility of this proposal for screening and diagnosis of pregnancy complications in the clinical routine.
